# Maternal X chromosome pericentric inversion resulting in the genetic analysis of offspring pedigrees with deletions at Xp22.33 and Xp22.33p11.3, and duplications at Xq27.3q28: Case report

**DOI:** 10.1097/MD.0000000000041255

**Published:** 2025-01-10

**Authors:** Guo-Sheng Deng, Yu-Qing Lai, Bo-Wen Luo, Yu-Di Luo, Ling-Ling Zhu, Zeng-Yu Yang, Keng Feng, De-Rong Li, Xiang Li

**Affiliations:** a Clinical Laboratory, Yulin Maternal and Child Health Care Hospital, Yulin, Guangxi, China; b Perinatal Health Care Department, Yulin Maternal and Child Health Care Hospital, Yulin, Guangxi, China; c Reproductive Medicine Center, Yulin Maternal and Child Health Care Hospital, Yulin, Guangxi, China.

**Keywords:** case report, duplications at Xq27.3q28, genetic counseling, maternal X chromosome pericentric inversion, pedigree analysis

## Abstract

**Rationale:**

This study investigates the genetic cause of primary infertility and short stature in a woman, focusing on maternal X chromosome pericentric inversion and its impact on offspring genetic outcomes, including deletions at Xp22.33 and Xp22.33p11.3, and duplications spanning Xq27.3 to the distal end of the X chromosome’s long arm.

**Patient concerns:**

The proband presented with primary infertility, menstrual irregularities, and ultrasound findings indicating a small uterus.

**Diagnoses:**

Peripheral blood G-banded karyotype analysis and single nucleotide polymorphism array analysis revealed a 46,X,rec(X)dup(Xq)inv(X)(p11.3q27)dmat karyotype in the proband, inherited from her mother. Genetic testing identified pathogenic deletions at Xp22.33 and Xp22.33p11.3, and a pathogenic duplication at Xq27.3q28.

**Interventions:**

Genetic counseling and pedigree analysis were conducted to trace the maternal origin of the pericentric inversion and assess recurrence risks.

**Outcomes:**

The study confirmed the maternal X chromosome pericentric inversion caused the observed genetic abnormalities, with a 50% recurrence risk for X-linked inheritance.

**Lessons:**

Maternal X chromosome pericentric inversion significantly affects offspring genetic outcomes. Assisted reproductive technologies, including in vitro fertilization with preimplantation genetic testing, are recommended to reduce recurrence risks in future pregnancies. Prenatal genetic testing is advised for natural conception to ensure fetal genetic health.

## 1. Introduction

Pericentric inversion is a chromosomal rearrangement in which both arms of a chromosome break simultaneously, and the middle segment reattaches in an inverted orientation to the end segments. Carriers of pericentric inversions form unique inversion loops during meiosis, resulting in the production of unbalanced gametes due to abnormal exchanges within the inversion loop. Given the unique nature of the X chromosome, the phenotypic effects of an inverted X chromosome depend on the breakpoints. Sarto et al suggested that breakpoints between X chromosome region spanning Xq13 to Xq26 (Xq13–26) may lead to infertility.^[1]^ Therman et al found that female carriers with intact Xq13–26 typically exhibit normal ovarian function.^[2]^ Associations occurring in the pseudoautosomal regions at the ends of the short arm of the X chromosome (Xp) and Yp generally do not affect meiosis in male carriers.^[3]^

In clinical practice, the cases we often encounter exhibit deletions in the Xp22.33 and Xp22.33p11.3 regions, and duplications in the region spanning Xq27.3 to Xq28 (Xq27.3q28) region resulting from X chromosome pericentric inversion. These deletions and duplications are closely linked to primary infertility and short stature arising from premature ovarian insufficiency.

This study aims to thoroughly investigate the transmission patterns, potential pathogenic mechanisms, and correlations with specific clinical phenotypes, of deletions in the Xp22.33p11.3 region and duplications in the Xq27.3q28 region resulting from pericentric inversion in pedigrees.

## 2. Case presentation

The proband, a 24-year-old female, has been experiencing primary infertility for the past 4 years. Her menstrual cycle typically spans approximately 40 days, with moderate flow lasting 5 to 7 days. She exhibits central obesity, weighing 65 kg with having a height of 145 cm, and displays well-developed secondary sexual characteristics without apparent abnormalities in the reproductive system and normal sexual function. On the third day of her menstrual cycle, she underwent a comprehensive hormonal examination at our hospital, which yielded results within the normal range. Her anti-Müllerian hormone level was 0.21 ng/mL, falling below the reference value. In May 2020, she decided to undergo assisted reproductive technology at our Reproductive Center due to primary infertility. Peripheral blood chromosome analysis and chromosomal microarray analysis were performed for the proband, her husband, and her parents. The proband has 2 sisters and 1 brother, all with normal phenotypes, none of whom have undergone chromosome analysis due to their unmarried status. The proband’s mother has no history of miscarriage and exhibits a normal phenotype. This study was approved by the Ethics Committee of Yulin Maternal and Child Health Care Hospital (Approval Number: YLSFYLLKY2024-1-22-01). All participants signed informed consent forms prior to participation, indicating their voluntary involvement and understanding of the study’s purpose, procedures, and potential risks and benefits.

### 2.1. Sample collection

The proband’s blood sample consisted of 3 mL of heparin anticoagulated blood and 2 mL of ethylenediaminetetraacetic acid-anticoagulated blood, intended for routine chromosomal karyotyping and chromosomal microarray analysis. Concurrently, peripheral blood samples were collected from the proband’s spouse and parents for chromosomal karyotyping.

### 2.2. Chromosomal G-banding karyotyping analysis

Peripheral blood samples were collected from the proband, her husband, father, and mother for chromosomal karyotyping. 0.3 mL of heparin-anticoagulated blood from each sample was cultured in AB lymphocyte culture medium using both conventional and synchronized culture methods. The conventional culture method included adding colchicine (20 μg/mL) 3 hours before harvest, while the synchronized method involved sequential addition of 5-fluorouracil, thymidine, and colchicine (100 μg/mL) during culture.

Chromosomes were prepared using G-banding techniques, and metaphase spreads were analyzed with an automatic scanning microscope and artificial intelligence-based image analysis software. Karyotypes of 30 cells were counted, and 5 cells were analyzed in detail according to the International System for Human Cytogenomic Nomenclature.^[4]^

### 2.3. Single nucleotide polymorphism array (SNP array) analysis

The CytoScan 750K Array Kit (Affymetrix, Santa Clara, CA) was used, which contains approximately 750,000 copy number probes and 200,000 SNP probes, designed to cover known oncogenes. The results were analyzed using software compatible with the Affymetrix microarray platform and compared with relevant databases. Interpretation of results: Copy number variants were classified into 5 categories based on established guidelines: pathogenic, likely pathogenic, uncertain clinical significance, likely benign, and benign.

## 3. Results

### 3.1. Chromosomal karyotype analysis

**Figure 1.**
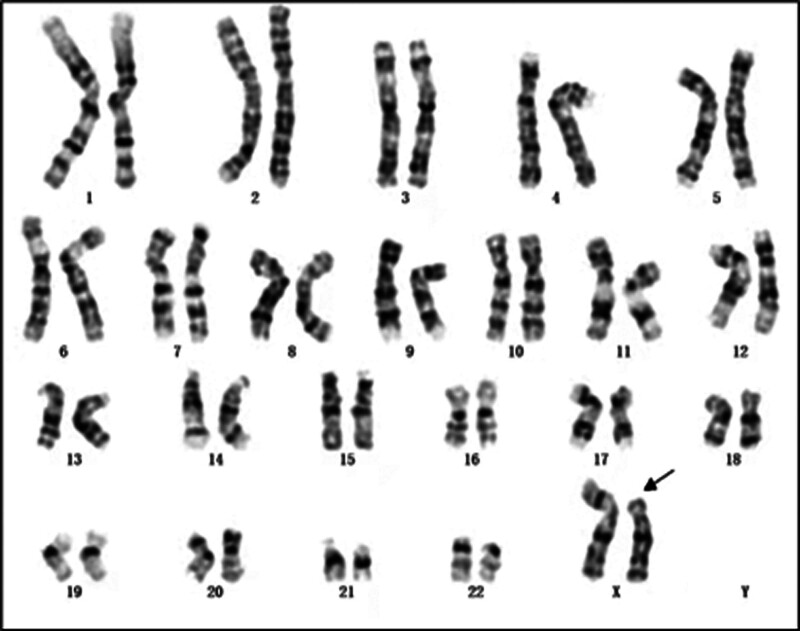
Peripheral blood chromosomal karyotype of the proband: 46,X,rec(X)dup(Xq)inv(X)(p11.3q27)dmat.

**Figure 2.**
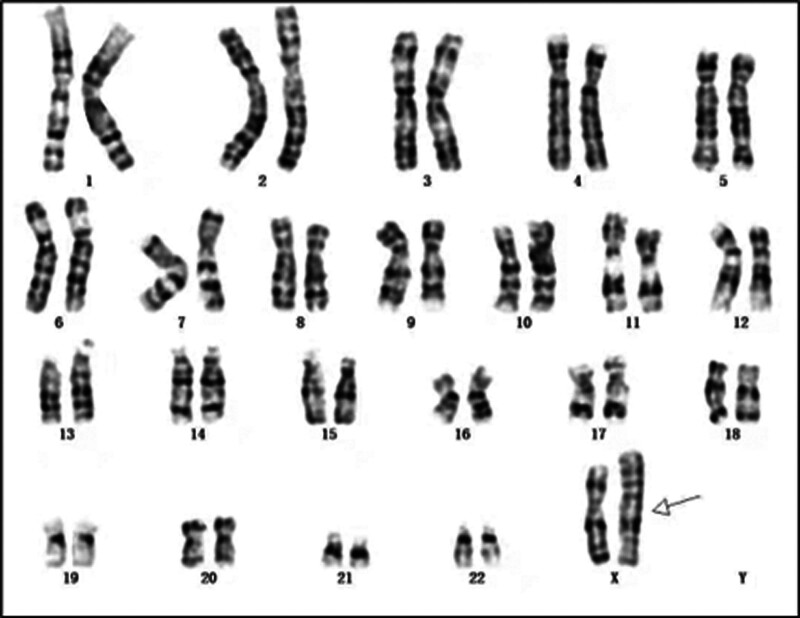
Peripheral blood chromosomal karyotype of the proband’s mother: 46,X,inv(X)(p11.3q27).

The proband’s chromosomal karyotype was identified as 46,X,rec(X)dup(Xq)inv(X)(p11.3q27)dmat, indicating a recombinant X chromosome derived from her mother. This result is depicted in Figure 1. The mother’s karyotype was 46,X,inv(X)(p11.3q27), revealing an X chromosome pericentric inversion, as shown in Figure 2. No chromosomal abnormalities were identified in the karyotypes of the proband’s husband and father, confirming they did not carry related genetic variations.

### 3.2. SNP array analysis of the proband

**Figure 3.**
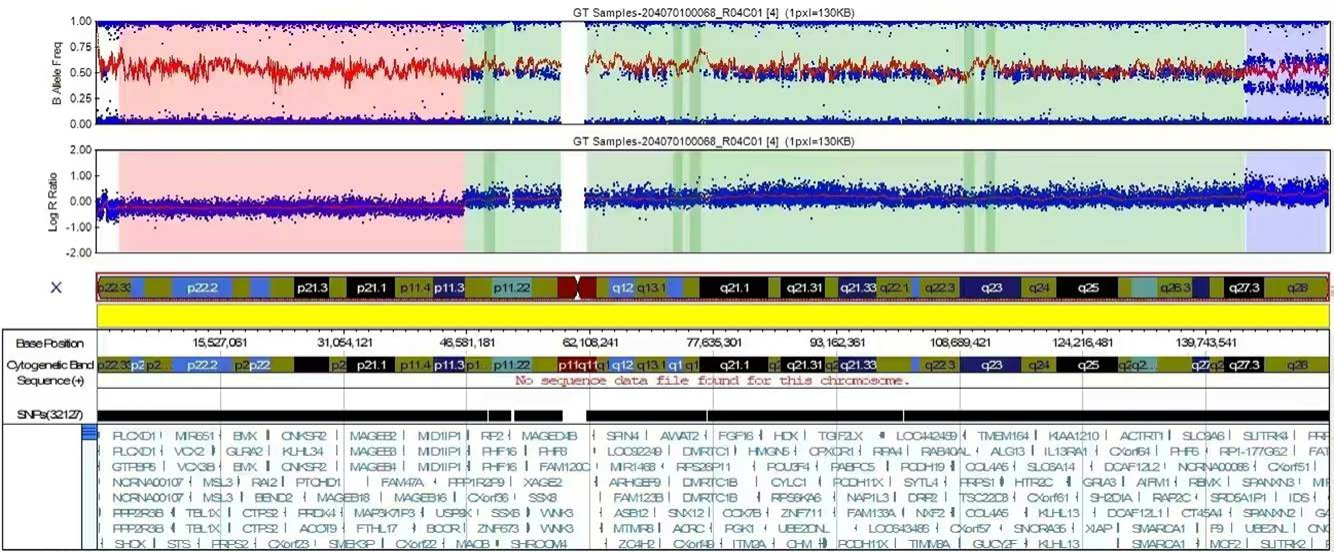
SNP array analysis results of the proband. SNP array = single nucleotide polymorphism array.

Peripheral blood SNP array analysis of the proband revealed the following: (1) A pathogenic deletion of 0.81 Mb in the pseudoautosomal region of the X chromosome at p22.23. (2) A pathogenic deletion spanning 44.90 Mb from Xp22.33 to Xp11.3 on the X chromosome, known to be associated with several clinical phenotypes. (3) A pathogenic duplication of 10.50 Mb in the X chromosome region extending from Xq27.3 to region at the distal end of the X chromosome’s long arm (Xq28), which includes genes implicated in intellectual disability and developmental disorders (Fig. 3).

### 3.3. Family investigation of the proband

**Figure 4.**
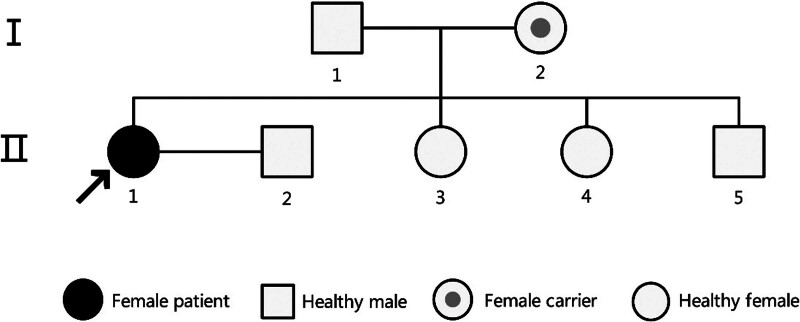
Results of the proband’s family investigation.

The extensive deletions and duplications detected on the proband’s X chromosome through SNP array analysis prompted further investigation to trace their origin via parental chromosomal karyotyping. The family pedigree is illustrated in Figure 4. The proband (II1) inherited the recombinant X chromosome from her mother (I2), who has no history of miscarriage and exhibits a normal phenotype. The father (I1) is in good health and shows a normal phenotype. The proband has 2 sisters and 1 brother, all of whom present with normal phenotypes. However, chromosomal testing has not been performed on her siblings as they are unmarried and have not undergone clinical evaluation.

### 3.4. Comprehensive genetic analysis and clinical implications

Through a comprehensive analysis of chromosomal karyotyping, genetic testing, and pedigree investigation, the proband was identified as carrying a recombinant X chromosome: 46,X,rec(X)dup(Xq)inv(X)(p11.3q27)dmat, which was maternally inherited. The deletions in the Xp22.33 and Xp22.33p11.3 regions, along with the copy number variation in the Xq27.3q28 region, were classified as pathogenic.

The deletion in the Xp22.33p11.3 region is associated with a wide spectrum of clinical manifestations, including cataracts, mild growth retardation, premature ovarian insufficiency, mild intellectual disability, central obesity, short stature, delayed motor development, facial abnormalities, autism, and joint contractures. This region contains several Online Mendelian Inheritance in Man-listed pathogenic genes, such as steroid sulfatase, dystrophin, and neuroligin 4 (NLGN4X), which are X-linked. Previous studies have highlighted phenotypic heterogeneity in individuals with deletions in Xp, with common presentations including growth retardation, short stature, and gonadal dysgenesis.

The duplication in the Xq27.3q28 region has been linked to “Xq28 Methyl-CpG-binding protein 2 duplication syndrome” and “Xq28 duplication syndrome.” The Xq28 duplication syndrome is an X-linked disorder associated with intellectual disability, cognitive impairment, attention-deficit hyperactivity disorder, behavioral abnormalities, recurrent infections, obesity, and infantile hypotonia in males. Female carriers generally exhibit milder phenotypes, which may include learning difficulties, facial anomalies, and cognitive impairments. The key clinical features often encompass intrauterine and postnatal growth retardation, facial anomalies, microcephaly, constipation, delayed language development, hypotonia, specific learning disabilities, and cognitive challenges.

Given the proband’s low anti-Müllerian hormone levels, premature ovarian insufficiency, and the 50% recurrence risk of X-linked inheritance within the family, it is strongly recommended to utilize assisted reproductive technologies such as in vitro fertilization with preimplantation genetic testing (PGT) for future pregnancies. If natural conception occurs, prenatal genetic testing is advised to ensure the genetic health of the fetus.

## 4. Discussion

The SNP array results of the proband revealed pathogenic variants, specifically deletions in the Xp22.33 and Xp22.33p11.3 regions, alongside a duplication in the Xq27.3q28 region. Given the chromosomal karyotype of the proband’s mother, 46, X,inv(X)(p11.3q27), we inferred the proband’s karyotype to be 46,X,rec(X)dup(Xq)inv(X)(p11.3q27)dmat, based on the integrated findings from the SNP array. The strongly suggests that the maternal X chromosome pericentric inversion induces deletions in the Xp22.33 and Xp22.33p11.3 regions, along with the duplication in the Xq27.3q28 region.

**Figure 5.**
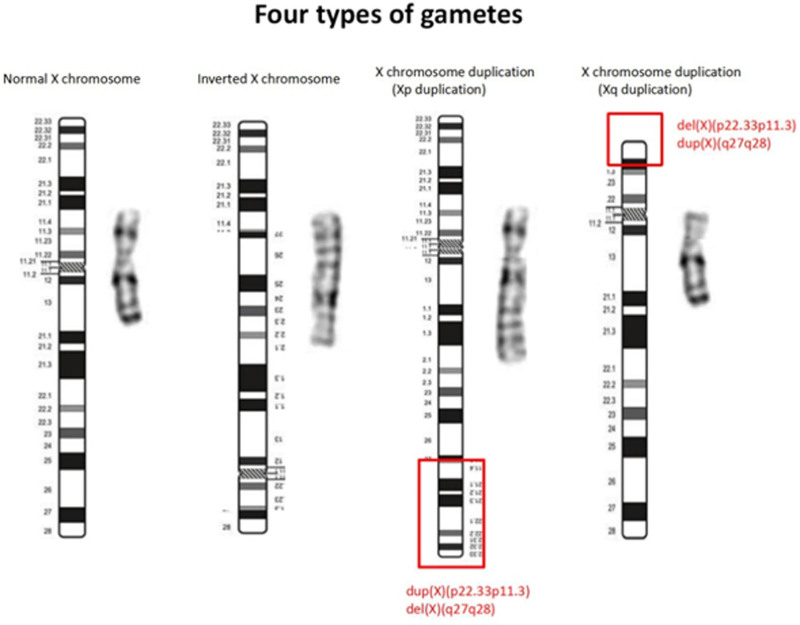
Four types of gametes formed by X chromosome pericentric inversion.

Individuals carrying X chromosome pericentric inversion typically exhibit a normal clinical phenotype, as no gene loss occurs. However, during meiosis I, when homologous chromosomes pair, a distinct inversion loop forms. The occurrence of crossover within this loop depends on the breakpoint locations, the inverted segment length, and the specific chromosome involved. Theoretically, when the number of exchanges within the inversion loop is odd, 4 different gametes will result (as illustrated in Fig. 5): 1 with a normal chromosome, 1 with an inverted chromosome, and the remaining 2 with rearranged chromosomes harboring partial duplications and deletions. Among these unbalanced rearranged chromosomes, 1 X chromosome has a duplication in the Xq27 to terminal region of the X chromosome long arm region and deletion from terminal region of the X chromosome short arm to Xp11.3, while the other X chromosome has a deletion in the Xq27 to terminal region of the X chromosome long arm region and a duplication in the terminal region of the X chromosome short arm to Xp11.3 region.^[5]^ When fertilized by sperm carrying a normal X chromosome, a female with a karyotype of 46,X,rec(X)dup(Xq)inv(X)(p11.3q27) is conceived, consistent with the proband’s chromosomal karyotype. Since this abnormal chromosome has only 1 centromere, it’s considered a stable aberration that doesn’t disrupt early embryo mitosis. Its genetic effects mainly depend on the length of the duplicated and segments and the lethal effects of the contained genes. Generally, a shorter inverted segment correlates with larger duplicated and deleted parts, reducing the likelihood of normal gamete and zygote development. Clinically, this may manifest as infertility, prolonged menstrual cycles, early miscarriages, stillbirths, and reduced chances of delivering malformed infants. Conversely, a longer inverted segment correlates with shorter duplicated and deleted regions, increasing the likelihood of normal gamete and zygote development, but also raising the risk of delivering malformed fetuses.^[6]^

Carriers of X chromosome pericentric inversion may experience infertility or adverse pregnancy outcomes. However, this particular carrier has successfully delivered 3 daughters and 1 son, with no history of adverse pregnancy outcomes. Sarto et al^[7]^suggested that breakpoints within the Xq13–26 region could cause infertility, while Therman et al^[2]^ proposed that women with an intact Xq13–26 region generally exhibit normal ovarian function. In male carriers, the inverted segment of the X chromosome typical does not pair with the Y chromosome during meiosis, thus minimally affecting meiosis and maintaining normal spermatogenesis.^[3]^ Due to the unique characteristics of the X chromosome, the genetic effects of X chromosome pericentric inversion include: (1) Breakpoints in specific X chromosome regions, such as critical areas, may affect female phenotypes. For instance, if the X chromosome’s long arm breakpoint falls within critical regions like Xq13-q22 or Xq22-q26, it could lead to gonadal dysfunction, primary amenorrhea, or premature ovarian insufficiency, although normal reproductive function remains possible. (2) There is a risk of having daughters with recombinant X chromosomes, although the likelihood of X chromosome recombination is lower than that of autosomal inversions. Based on the segments deleted from either Xp or the long arm of the X chromosome (Xq) due to recombination, certain abnormalities can be predicted. For example, deletion of Xp may lead to short stature, while deletion of Xq may result in ovarian failure. (3) Significant gender differences exist during reproduction of carriers of X chromosome pericentric inversion.

In this investigation, the genetic chip analysis detected a 0.81 Mb deletion in the X chromosome pseudoautosomal region (Xp22.33) of the X chromosome in the proband. This deletion encompasses the disease locus associated with “Leri-Weill Dyschondrosteosis-Short Stature Homeobox gene defect syndrome.” Patients with *short stature homeobox* gene defects manifest a wide array of clinical features, including disproportionate short stature, restricted forward or backward rotation of the forearms, Madelung deformity, and radial curvature. Additionally, a 44.90 Mb deletion was identified in the X chromosome region p22.33p11.3, covering the disease locus related to “Steroid Sulfatase Deficiency (*STS*)" (X:6455812-8133195, approximately 1.68 Mb).

Notably, the Database of Chromosomal Imbalance and Phenotype in Humans using Ensembl Resources and International Standards for Cytogenomic Arrays document numerous pathogenic cases linked to smaller deletions within this segment, with prominent clinical manifestations, including cataracts, mild growth retardation, premature ovarian insufficiency, mild intellectual disability, central obesity, short stature, delayed motor development, facial abnormalities, autism, and contractures. This genomic region harbors several Online Mendelian Inheritance in Man pathogenic genes, including *steroid sulfatase*, *dystrophin*, and *NLGN4X*. Literature suggests that X chromosome short arm deletions exhibit phenotypic heterogeneity, possibly associated with growth retardation, short stature, and gonadal dysgenesis.^[8]^

Furthermore, a 10.50 Mb duplication was identified in the X chromosome region q27.3q28, encompassing the disease loci of “Xq28 (*Methyl-CpG-binding Protein 2*) duplication syndrome” (X:153287263-153363188, approximately 75.9 Kb) and “Xq28 duplication syndrome” (X:153624563-153881853, approximately 257.3 Kb). “Xq28 duplication syndrome” is characterized by X-linked intellectual disability, with male patients experiencing intellectual disability, cognitive impairment, attention-deficit hyperactivity disorder, behavioral abnormalities, recurrent infections, obesity, and infantile hypotonia. Female patients exhibit milder clinical phenotypes, including learning difficulties, facial anomalies, and cognitive impairment.^[9]^ Both Database of Chromosomal Imbalance and Phenotype in Humans using Ensembl Resources databases and International Standards for Cytogenomic Arrays document multiple pathogenic cases associated with duplications smaller than this segment, presenting major clinical symptoms such as intrauterine/postnatal growth retardation, facial anomalies, microcephaly, constipation, delayed language development, hypotonia, specific learning disabilities, and cognitive impairment.

The proband’s mother possesses an X chromosome inversion segment spanning Xp11→Xq27, circumventing the critical Xq13–26 interval and enabling normal reproductive function. However, the proband’s recombinant X chromosome, which features deletions in the Xp22.33 and Xp22.33p11.3 segments, leads to conditions such as stature and premature ovarian insufficiency, culminating in primary infertility.^[10]^ The effects of the Xq segment duplication mitigate these consequences through selective inactivation, resulting in the manifestation of only the gene effects associated with Xp deletion, such as short stature and premature ovarian insufficiency, consistent with literature findings.^[11]^

In females carrying X chromosome pericentric inversion, the inverted X chromosome undergoes recombination during meiosis in their oocytes. If an oocyte contains a recombinant X chromosome, the clinical phenotype of the resulting embryo depends on whether the fertilizing sperm carries an X or Y chromosome. In embryos with a karyotype of 46,X,rec(X), girls m present with the following phenotypes: (1) Xq deletion and Xp duplication, leading to normal or tall stature but ovarian dysfunction. (2) Xp deletion and Xq duplication, resulting in short stature but normal ovarian function. The concurrent X chromosome duplication can mitigate these effects through selective inactivation, predominantly exhibiting the gene effects of Xp deletion. In embryos with a karyotype of 46,Y,rec(X), the outcomes include: (1) Deletion of a segment of X chromosome, resulting in a deletion chromosome. If this deletion encompasses more than the smallest chromosomal segments, the embryo is not viable. (2) Deletion of a small terminal segment, where male embryos may survive but typically exhibit severe intellectual disabilities and malformations, often leading to lethality.^[12]^

Given the unique characteristics of the X chromosome, the genetic implications of pericentric inversion are more complex compared to autosomes inversions. Accurate genetic counseling for carriers of X chromosome pericentric inversion is essential, as it plays a significant role in patient decision-making.

For this proband, PGT is strongly recommended due to the 50% recurrence risk of X-linked inheritance within the family. PGT allows for the selection of embryos free from chromosomal abnormalities before implantation, thereby reducing the likelihood of passing on the identified pathogenic variations. Additionally, if the proband conceives in the future, prenatal diagnostic testing, such as chorionic villus sampling or amniocentesis, is essential to confirm the chromosomal status of the fetus. These measures are critical for ensuring informed reproductive choices and optimizing clinical outcomes for the proband and her offspring.

This study has several limitations. First, it is based on a single case and a small sample size, which limits the generalizability of the findings. The proband’s immediate family members were tested, but the lack of broader family genetic testing, particularly for the proband’s siblings, restricts our ability to fully assess inheritance patterns and genetic variability within the extended family. Additionally, the study lacks long-term follow-up data on the proband’s reproductive outcomes and health status, which could provide further insights into the long-term effects of the identified genetic variations. Moreover, although pathogenic deletions and duplications were identified, the clinical manifestations associated with these genetic changes are not fully established in the literature, particularly in females with X chromosome duplications. Lastly, the use of advanced genomic technologies, such as G-banded karyotyping and SNP array analysis, may miss smaller genetic alterations, and clinical features associated with these chromosomal anomalies may not have been fully captured.

Future studies with larger sample sizes, extended family analysis, and long-term clinical follow-up would help address these limitations and provide a more comprehensive understanding of the genetic implications and recurrence risks for patients with similar chromosomal abnormalities.

## 5. Conclusion

In this study, we conducted a thorough analysis of the chromosomal karyotypes and conducted SNP array testing in the offspring of a maternal X chromosome pericentric inversion. This inversion resulted in deletions in the Xp22.33 and Xp22.33p11.3 regions, as well as duplications in the Xq27.3q28 region. We explored the relationship between the proband’s clinical phenotype and genotype, thereby deepening our understanding of the genetic alterations involved. Given these genomic changes, along with the inheritance patterns of the X chromosome, the recurrence risk for the proband’s offspring is estimated to be as high as 50%. Therefore, we recommend the use of assisted reproductive technologies, such as in vitro fertilization and PGT, to reduce the likelihood of passing on these genetic variations in future pregnancies. Additionally, in the case of a natural pregnancy, we advise prenatal genetic testing to ensure the health of the fetus.

## Acknowledgments

We would like to thank Yulin Maternal and Child Health Care Hospital for their specific contributions, such as assistance with data collection, technical support, or helpful discussions.

## Author contributions

**Conceptualization:** Guo-Sheng Deng, Yu-Qing Lai, Yu-Di Luo, Zeng-Yu Yang, De-Rong Li, Xiang Li.

**Data curation:** Guo-Sheng Deng, Yu-Qing Lai, Bo-Wen Luo, Ling-Ling Zhu, Zeng-Yu Yang, Xiang Li.

**Formal analysis:** Ling-Ling Zhu, Zeng-Yu Yang.

**Funding acquisition:** Ling-Ling Zhu, Zeng-Yu Yang, De-Rong Li, Xiang Li.

**Investigation:** Keng Feng.

**Methodology:** Keng Feng.
